# Integrative Analysis of Brain Region-specific Shank3 Interactomes for Understanding the Heterogeneity of Neuronal Pathophysiology Related to *SHANK3* Mutations

**DOI:** 10.3389/fnmol.2017.00110

**Published:** 2017-04-19

**Authors:** Yeunkum Lee, Hyojin Kang, Bokyoung Lee, Yinhua Zhang, Yoonhee Kim, Shinhyun Kim, Won-Ki Kim, Kihoon Han

**Affiliations:** ^1^Department of Neuroscience, College of Medicine, Korea UniversitySeoul, South Korea; ^2^Department of Biomedical Sciences, College of Medicine, Korea UniversitySeoul, South Korea; ^3^HPC-enabled Convergence Technology Research Division, Korea Institute of Science and Technology InformationDaejeon, South Korea

**Keywords:** Shank3, interactome, mPFC, striatum, hippocampus

## Abstract

Recent molecular genetic studies have identified 100s of risk genes for various neurodevelopmental and neuropsychiatric disorders. As the number of risk genes increases, it is becoming clear that different mutations of a single gene could cause different types of disorders. One of the best examples of such a gene is *SHANK3*, which encodes a core scaffold protein of the neuronal excitatory post-synapse. Deletions, duplications, and point mutations of *SHANK3* are associated with autism spectrum disorders, intellectual disability, schizophrenia, bipolar disorder, and attention deficit hyperactivity disorder. Nevertheless, how the different mutations of *SHANK3* can lead to such phenotypic diversity remains largely unknown. In this study, we investigated whether Shank3 could form protein complexes in a brain region-specific manner, which might contribute to the heterogeneity of neuronal pathophysiology caused by *SHANK3* mutations. To test this, we generated a medial prefrontal cortex (mPFC) Shank3 *in vivo* interactome consisting of 211 proteins, and compared this protein list with a Shank3 interactome previously generated from mixed hippocampal and striatal (HP+STR) tissues. Unexpectedly, we found that only 47 proteins (about 20%) were common between the two interactomes, while 164 and 208 proteins were specifically identified in the mPFC and HP+STR interactomes, respectively. Each of the mPFC- and HP+STR-specific Shank3 interactomes represents a highly interconnected network. Upon comparing the brain region-enriched proteomes, we found that the large difference between the mPFC and HP+STR Shank3 interactomes could not be explained by differential protein expression profiles among the brain regions. Importantly, bioinformatic pathway analysis revealed that the representative biological functions of the mPFC- and HP+STR-specific Shank3 interactomes were different, suggesting that these interactors could mediate the brain region-specific functions of Shank3. Meanwhile, the same analysis on the common Shank3 interactors, including Homer and GKAP/SAPAP proteins, suggested that they could mainly function as scaffolding proteins at the post-synaptic density. Lastly, we found that the mPFC- and HP+STR-specific Shank3 interactomes contained a significant number of proteins associated with neurodevelopmental and neuropsychiatric disorders. These results suggest that Shank3 can form protein complexes in a brain region-specific manner, which might contribute to the pathophysiological and phenotypic diversity of disorders related to *SHANK3* mutations.

## Introduction

Recent genome-wide molecular genetic studies on human patients have identified 100s of risk genes for neurodevelopmental and neuropsychiatric disorders including autism spectrum disorders (ASDs), intellectual disability, schizophrenia (SCZ), bipolar disorder (BD), and depression. An interesting finding from these studies is that many risk genes are shared by multiple disorders (Lichtenstein et al., 2010; Zhu et al., 2014). In other words, different mutations in the same gene can cause or contribute to different types of disorders. One of the best examples of such genes is *SHANK3* (for SH3 and multiple ankyrin repeat domains 3, also called *ProSAP2* for proline-rich synapse-associated protein 2), which encodes a core scaffold protein organizing the macromolecular protein complex of the neuronal excitatory post-synapse (Sheng and Kim, 2000; Dosemeci et al., 2016). Deletions of the chromosomal region containing *SHANK3* cause Phelan-McDermid syndrome (22q13 deletion syndrome) characterized by autistic behaviors, intellectual disability, and epilepsy (Wilson et al., 2003). Variety of point mutations and small deletions in the *SHANK3* gene have been found in patients with ASDs, intellectual disability, and SCZ (Durand et al., 2007; Gauthier et al., 2010; Grabrucker et al., 2011; Jiang and Ehlers, 2013; Guilmatre et al., 2014; Leblond et al., 2014). Moreover, duplications of the *SHANK3* gene have also been identified in patients with Asperger’s syndrome, BD, SCZ, and attention deficit hyperactivity disorder (ADHD) (Failla et al., 2007; Moessner et al., 2007; Han et al., 2013; Choi and Han, 2015). Importantly, many of the risk genes for neurodevelopmental and neuropsychiatric disorders are involved in regulating synaptic development and function (van Spronsen and Hoogenraad, 2010; Zoghbi and Bear, 2012; Hall et al., 2015). As Shank3 is an abundant core synaptic protein, studying *SHANK3* gene as a leading case can provide some insight into the mechanisms by which different variants of a single gene, especially one functioning at the synapse, can lead to phenotypic diversity.

The neurobiological basis explaining how *SHANK3* mutations can lead to a range of disorders remains largely unknown. Nevertheless, recent molecular and animal model studies have provided some information that can help us understand the symptom heterogeneity. First, human *SHANK3* and rodent *Shank3* genes express many Shank3 isoforms due to alternative splicing and the presence of multiple internal promoters (Jiang and Ehlers, 2013). Therefore, different mutations in the *SHANK3* gene can affect different subsets of isoforms (Jiang and Ehlers, 2013). This was supported by some phenotypic differences observed among *Shank3* “partial” knock-out (KO) mice generated by deletions of different exons of the gene (Bozdagi et al., 2010; Peca et al., 2011; Wang et al., 2011; Schmeisser et al., 2012; Yang et al., 2012; Kouser et al., 2013; Lee et al., 2015; Speed et al., 2015). Second, each brain region expresses different groups and levels of Shank3 isoforms (Wang et al., 2014). Therefore, the pathophysiology of brain regions may vary according to the *SHANK3* mutation. Indeed, even in a single *Shank3* KO mouse, the biochemical and electrophysiological defects of neurons in the hippocampus (HP), striatum (STR), and medial prefrontal cortex (mPFC) can vary (Peca et al., 2011; Lee et al., 2015). Importantly, Zhou et al. (2016) recently demonstrated that two different *Shank3* knock-in (KI) mice modeling the human mutations of ASD (InsG3680) and SCZ (R1117X) display STR- and PFC-specific synaptic defects, respectively. Third, compensatory roles of other Shank family members, Shank1 and Shank2 (Schmeisser et al., 2012), and regulatory molecules of *SHANK3* expression such as microRNAs (Choi et al., 2015) in some brain regions could be also considered.

Another relatively unexplored mechanism that might underlie differences among brain regions involves the protein–protein interactions (PPIs) of Shank3. The Shank3 protein harbors multiple PPI domains such as SPN, ankyrin repeats, SH3, PDZ, proline-rich, and SAM domains from the N-terminus to the C-terminus (Sheng and Kim, 2000; Mameza et al., 2013). So far, 100s of proteins directly or indirectly interacting with Shank3 through the PPI domains have been identified from both *in vitro* and *in vivo* studies (Sakai et al., 2011; Han et al., 2013), and the functional relationships between some of these proteins and Shank3 have been characterized (Sheng and Kim, 2011; Guilmatre et al., 2014). Notably, a recent quantitative proteomic study revealed differential protein expression profiles across mouse brain regions (Sharma et al., 2015). Moreover, it has been demonstrated that AMPA (α-amino-3-hydroxy-5-methyl-4-isoxazolepropionic acid)-type glutamate receptors at the excitatory post-synapse form brain region-specific protein complexes, which is critical for differential and sophisticated regulation of AMPA receptors in each brain region (Chen et al., 2014; Schwenk et al., 2014). Therefore, it is also conceivable that Shank3 might have brain region-specific interactomes, which, together with the above-mentioned factors, could contribute to the phenotypic complexity and heterogeneity of *SHANK3* related disorders. However, so far whether and, if so, what kinds of proteins can interact with Shank3 in a brain region-specific manner have not been investigated.

We have previously generated a Shank3 interactome by combining results from yeast two-hybrid (Y2H) screening (Sakai et al., 2011) and *in vivo* immunoprecipitation (IP) followed by mass spectrometry analysis of the mixed HP and STR (HP+STR) tissue (Han et al., 2013). In this study, we newly generated a mPFC Shank3 *in vivo* interactome. Together with our previous data, we identified the common proteins interacting with Shank3 across brain regions, or specifically in the mPFC or HP+STR. We also constructed interactome networks from the protein lists and characterized their properties. Furthermore, we performed a variety of bioinformatic analysis to understand the representative biological functions and disease associations of each brain region-specific Shank3 interactome. From this integrative analysis, we propose a hypothesis that the brain region-specific Shank3 interactomes might contribute to the heterogeneity of neuronal pathophysiology and the diversity of phenotypes related to *SHANK3* mutations.

## Materials and Methods

### Mice

The *EGFP* (enhanced green fluorescent protein)*-Shank3* transgenic (TG) mice used in this study have been described previously (Han et al., 2013). The wild-type (WT) and TG mice were bred and maintained in a C57BL/6J background according to the Korea University College of Medicine Research Requirements, and all the experimental procedures were approved by the Committees on Animal Research at Korea University College of Medicine (KOREA-2016-0096). The mice were fed *ad libitum* and housed under a 12-h light–dark cycle.

### Immunoprecipitation and Mass Spectrometry

The mice (WT and TG littermates at 5-week-old age) were deeply anesthetized with isoflurane and decapitated. The mPFC was dissected from each brain using brain matrix, immediately frozen in liquid nitrogen and stored at -80°C until its use for immunoprecipitation. The mPFC tissue was collected from 20 animals of each genotype. The mPFC tissue from 20 animals was pooled and homogenized in sucrose buffer (320 mM sucrose, 10 mM Tris-HCl, 5 mM EDTA, pH 7.4) with freshly added protease and phosphatase inhibitors (Roche). The P2 crude synaptosomal fraction (Han et al., 2009) of the mPFC was solubilized with DOC buffer (1% sodium deoxycholate, 50 mM Tris-HCl, pH 9.0), dialyzed against binding/dialysis buffer (50 mM Tris-HCl, pH 7.4, 0.1% Triton X-100), and centrifuged as previously described (Choi et al., 2005; Han et al., 2013). For the immunoprecipitation, 7 mg of the P2 DOC lysates, which was almost entire amount of lysates obtained from the mPFC pooled from 20 animals, was incubated with GFP-Trap beads (ChromoTek) for 2 h at 4°C. Therefore, the sample number of our mass spectrometry analysis was one for each genotype. Next, the beads were briefly washed with binding/dialysis buffer and boiled with 1x NuPAGE LDS sample buffer (Invitrogen) containing 1x NuPAGE reducing agent (Invitrogen). The eluted proteins were visualized using Coomassie Brilliant Blue. The gel pieces were destained and subjected to in-gel digestion using trypsin. Tryptic peptide was dissolved in the loading solution (99.9% water with 0.1% formic acid) and analyzed using the nanoflow LC–MS/MS system consisting of an Easy nLC1000 (Thermo Scientific) and an LTQ Orbitrap Elite mass spectrometer (Thermo Scientific, USA) equipped with a nano-electrospray source. The peptides were loaded onto a trap column (size, 20 mm × 75 μm) (C18, 3 μm from Thermo Scientific). Next, the trap column was washed with the loading solution and switched in-line with an in-house column (size, 200 mm × 100 μm) packed with a Reprosil-Pur 120C18-AQ (3 μm, Dr. Maisch GmBH). The peptides were separated with a 90 min discontinuous gradient of 5–50% acetonitrile/0.1% formic acid at a flow rate of 300 nl min^-1^. The separated peptides were directly electro-sprayed into LTQ Orbitrap Elite mass spectrometer. The LTQ Orbitrap instrument was operated in the data-dependent mode to acquire fragmentation spectra of the 15 strongest ions and under direct control of the Xcalibur software (Thermo Scientific). The obtained MS/MS spectra were searched against the target-decoy mouse refseq database (Uniprot mouse database – March 6, 2016 released) in the IP2 (Integrated Proteomics) pipeline. The precursor mass tolerance was confined within 10 p.p.m. with a fragment mass tolerance of 0.6 Da; the number of missed cleavage was two. Trypsin was selected as the enzyme. Carbamidomethylation at cysteine was chosen as static modifications. Oxidation at methionine was chosen as variable modifications. The output data files were filtered and sorted to create the protein list using the DTASelect (Tabb et al., 2002), with two or more peptides assignments for protein identification. The assigned peptides were filtered with a false discovery rate (FDR) of 1%. All the 137 proteins detected only in the TG sample were included for further analysis. Of the 259 proteins detected both in the WT and TG samples, only the 74 proteins satisfying both criteria (two or more sequence counts in the TG sample compared to the WT sample, and at least twice the total intensity in the TG sample compared to the WT sample) were included for further analysis. Mass spectrometry was performed using nano LC-LTQ-Orbitrap Elite mass spectrometry at the Korea Basic Science Institute (Ochang Head quarter, Division of Bioconvergence Analysis). The raw data of mass spectrometry analysis was submitted to MassIVE (Accession: MSV000080657) and ProteomeXchage (Accession: PXD006133) database.

### Subcellular Fractionation

Subcellular brain fractions were prepared as described previously (Han et al., 2009, 2015). Briefly, mouse cortical tissues were homogenized in buffered sucrose solution (0.32 M sucrose, 4 mM HEPES, 1 mM MgCl_2_, 0.5 mM CaCl_2_, pH 7.3) with freshly added protease inhibitors. This homogenate (fraction H) was centrifuged at 900 *g* for 10 min (the resulting pellet is P1). The resulting supernatant was centrifuged again at 12,000 *g* for 15 min (the supernatant after this is S2). The pellet was resuspended in buffered sucrose and centrifuged again at 13,000 *g* for 15 min (the resulting pellet is P2, crude synaptosome).

### Western Blot and Antibodies

The P2 DOC lysates and IP samples were boiled with 1x NuPAGE LDS sample buffer (Invitrogen) containing 1x NuPAGE reducing agent (Invitrogen). The antibodies used for Western blotting included β-actin (Santa Cruz Biotechnology, sc-47778), GAPDH (Cell Signaling, #2118), GFP (abcam, ab290), GluA1 (Millipore, 04-855), GluA2 (Millipore, MA397), Homer (Santa Cruz Biotechnology, sc-20807), mGluR5 (Millipore, AB5675), NeuN (Millipore, MAB377), PSD-95 (Thermo Scientific, MA1-046), Shank3 (Santa Cruz Biotechnology, sc-30193), and WAVE1 (NeuroMab, 75-048). The Western blot images were acquired using the ChemiDoc Touch Imaging System (Bio-Rad), and quantified by ImageJ software.

### Bioinformatic Analysis

#### Construction of Shank3 Interactome Networks

To build interaction networks with the sets of Shank3 interactors (common, mPFC-specific, and HP+STR-specific), PPIs were adopted from the Agile Protein Interactomes DataServer (APID)^1^, which provides consolidated protein interactions from the primary interaction databases (BIND, BioGRID, DIP, HPRD, IntAct, and MINT) and from experimentally resolved 3D structures of protein complexes (Alonso-Lopez et al., 2016). The level 1 (all known interactions) data between human and mouse genes were included (last update: Jan 13, 2017). The network graphics were generated with Cytoscape (Shannon et al., 2003). To simplify the network, orphan nodes, defined as nodes without any suggested interaction from the APID among proteins in the interactome, were excluded from the graphics.

#### Network Topology Analysis of Shank3 Interactome Networks

The average path length, average number of links along the shortest paths for all possible pairs of proteins, and degree distribution, the probability of a node having k links, were calculated to measure the network topology of brain region-specific interactome networks. To test the significance of network properties, the empirical re-sampling approach was used. The same number of proteins from the mouse brain interactome (see below) was randomly selected and their average path length was calculated. This re-sampling was repeated 10,000 times and an empirical *P*-value was calculated (N_i_ = the number of sample whose average path length is smaller than the average path length of the Shank3 interactome; *P-*value = N_i_/10,000).

#### Mouse Brain Interactome

The mouse brain proteome data was obtained from the results of a recent study (Sharma et al., 2015), in which they performed mass spectrometry-based quantitative proteomic analysis of 10 brain regions (hippocampus, thalamus, brain stem, motor cortex, corpus callosum, striatum, cerebellum, olfactory bulb, prefrontal cortex, and optic nerve). In total, 8,780 mouse proteins were converted to human homologs using the HUGO Gene Nomenclature Committee (HGNC) database^2^. The PPIs among these mouse brain proteins were adopted from the APID as described above. The total number of nodes and edges of mouse brain interactome were 7,880 and 319,756 respectively.

#### Gene Ontology (GO) and Kyoto Encyclopedia of Genes and Genomes (KEGG) Pathway Analysis

The Gene Ontology (GO) and Kyoto Encyclopedia of Genes and Genomes (KEGG) pathway analysis were performed using the DAVID software (version 6.8) (Huang da et al., 2009). The sets of Shank3 interactors from the common, mPFC-specific, and HP+STR-specific interactomes were tested against a customized background from the mouse brain proteome (Sharma et al., 2015).

#### Disease Association Analysis

Gene-disease association data were retrieved from the PsyGeNET (Psychiatric disorders Gene association NETwork) database (last update: September, 2016) (Gutierrez-Sacristan et al., 2015), which contains information about psychiatric diseases and their associated genes integrated from the DisGeNET (Pinero et al., 2017) database, and data extracted from the literature by text mining, which has been further curated by domain experts. Additionally, ASD risk genes were obtained from the SFARI (Simons Foundation Autism Research Initiative) database (syndromic and category 3 or above)^3^, and FMRP (Fragile X mental retardation protein) target genes were downloaded from a previous publication (Darnell et al., 2011). The enrichment of disease-associated genes was tested using the hypergeometric distribution test. Hypergeometric *P*-values were calculated using the phyper (*q*: overlapped genes-1, *m*: brain region specific genes, *n*: mouse brain interactome – *m, k*: disease associated genes) function in R package, and were adjusted for multiple testing with the Benjamini and Hochberg test, as implemented in the Bioconductor’s *q*-value package. Diseases with adjusted *P*-values less than 0.05 were considered statistically significantly enriched.

## Results

### Generation of the mPFC Shank3 Interactome

**FIGURE 1:**
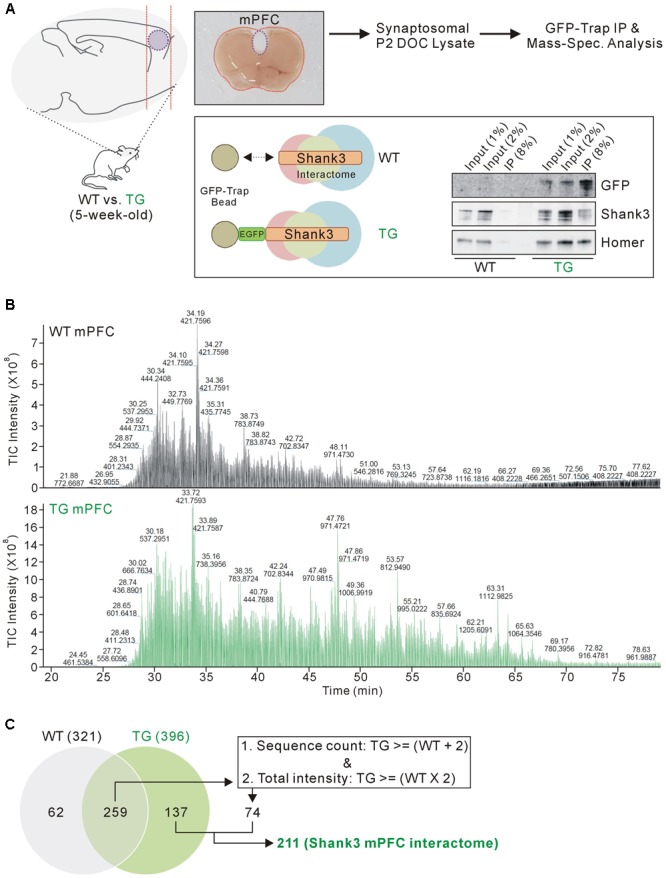
**Isolation and mass spectrometry analysis of the mPFC Shank3 protein complex. (A)** The diagram in the upper panel shows the experimental procedure. The mPFC (inside the purple dotted-line) was dissected from 5-week-old wild-type (WT) and *EGFP-Shank3* TG mice, as shown in the representative picture. The mPFC tissue from 20 animals of each genotype was collected and homogenized to produce synaptosomal (P2 fraction) deoxycholate (DOC) lysates. The protein complex bound to EGFP-Shank3 (interactome) was isolated using GFP-Trap beads (lower panel). Western blot images show that EGFP-Shank3 and its known binding partner Homer were pulled down only from the TG mPFC lysate. **(B)** Chromatograms of mass spectrometry analysis for the WT and TG IP samples. The numbers indicate retention time and intensity of each peak, respectively. TIC, total ion current. **(C)** The Venn diagram shows the number of proteins detected from the WT and TG IP samples. Of the 259 proteins detected in both the WT and TG IP samples, only those 74 proteins satisfying both criteria (two or more sequence counts in the TG IP sample compared to the WT IP sample, and at least twice the total intensity in the TG IP sample compared to the WT IP sample) were included for further analysis. Finally, 211 (137+74) proteins were considered to constitute the mPFC Shank3 *in vivo* interactome.

Previously, we generated a Shank3 *in vivo* interactome by performing IP followed by mass spectrometry analysis of the mixed HP and STR (HP+STR) synaptosomal P2 DOC lysates from 5-week-old *EGFP-Shank3* TG mice (Han et al., 2013). We repeated the same experiment using the mPFC tissues from 5-week-old *EGFP-Shank3* TG mice (**Figure 1A**). We confirmed that our preparation of synaptosomal P2 fraction could enrich post-synaptic proteins such as Shank3 and PSD-95, but exclude a nuclear protein NeuN (**Supplementary Figure S1**). The mPFC tissues from WT mice were used as a negative control for IP using the GFP-Trap beads that pulled down EGFP-Shank3 proteins only from the TG mPFC lysates (**Figure 1A**). Notably, in our Western blot experiments, the input to IP ratios of Shank3 proteins detected by GFP and Shank3 antibodies were different (**Figure 1A**). This could be due to the location of EGFP-tag in *EGFP-Shank3* transgene (the first start codon of *Shank3* gene, thus GFP-trap could pull down only the Shank3 isoforms expressed from the first promoter), the Shank3 antibody used (this antibody recognizes C-terminal domain, thus could miss some Shank3 isoforms without this domain), and/or some other factors need to be identified.

We selected the mPFC region for several reasons. First, the mPFC is involved in numerous cognitive functions including working memory, decision making, social cognition, and reward (Tzschentke, 2000; Euston et al., 2012; Grossmann, 2013), and anatomical and functional dysfunction of the mPFC has been identified in patients with various neurodevelopmental and neuropsychiatric disorders (Drevets et al., 2008; Chai et al., 2011), which could be also associated with *SHANK3* mutations. Second, Shank3 is highly expressed in the mouse mPFC (Lee et al., 2015). Third, the biochemical and electrophysiological changes in mPFC synapses were characterized in some *Shank3* KO and KI mice; these changes were different from the changes observed in HP and STR synapses in the same mice (Lee et al., 2015; Zhou et al., 2016). Taken together, we reasoned that the mPFC Shank3 interactome could be the primary candidate to be compared with the previously generated HP+STR Shank3 interactome. Similar to the HP and STR (Han et al., 2013), there was no significant difference in the expression levels of some synaptic proteins in the mPFC of *EGFP-Shank3* TG mice compared to WT mice (**Supplementary Figure S2**).

As expected, the chromatograms obtained from the mass spectrometry analysis showed that many more proteins were detected in the TG IP sample than in the WT IP sample (**Figure 1B**). The detection of proteins in the WT IP sample could possibly be due to non-specific binding to the GFP-Trap beads. When we matched the protein identities, 137 proteins were detected only in the TG IP sample, while 259 proteins were detected in both WT and TG IP samples (**Figure 1C**). Of the 259 proteins, we selected 74 proteins satisfying both criteria (two or more sequence counts in the TG IP sample compared to the WT IP sample, and at least twice the total intensity in the TG IP sample compared to the WT IP sample). Finally, 211 (137+74) proteins were considered to be part of the mPFC Shank3 *in vivo* interactome (**Figure 1C**).

### Construction and Characterization of Common and Brain Region-specific Shank3 Interactome Networks

**FIGURE 2:**
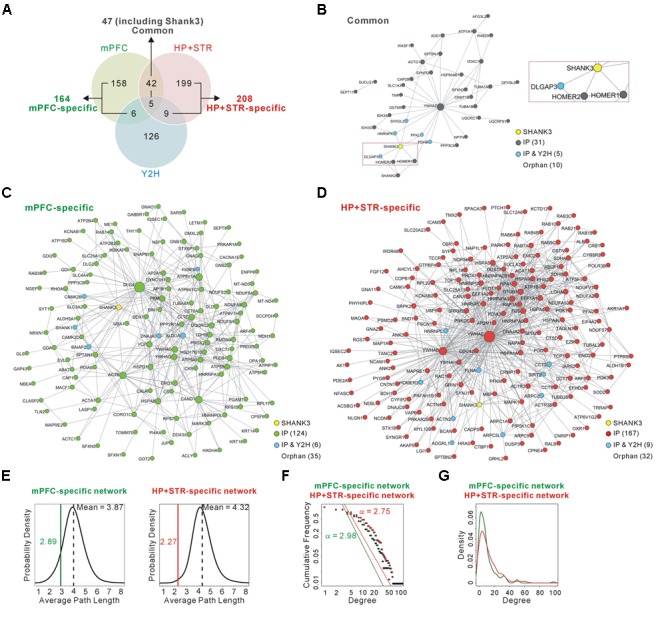
**Construction and characterization of the Shank3 interactome networks. (A)** The Venn diagram shows the number of Shank3-interacting proteins identified only in the mPFC (164), only in the HP+STR (208), or commonly in both the brain regions (47). Compared with the protein list from the Shank3 Y2H screening, only 11 and 14 proteins were shared by the mPFC and HP+STR interactomes, respectively. **(B)** The interactome network of common Shank3 binding partners (37 nodes and 57 edges) from the mPFC and HP+STR. To simplify the network, orphan nodes, defined as the nodes without any suggested interaction from the APID among the 47 proteins, were excluded from the network (10 orphan nodes). The DLGAP and Homer scaffold proteins are in the common interactome (inside the purple dotted-line). **(C)** The interactome network of mPFC-specific binding partners (131 nodes, 344 edges, and 35 orphan nodes). **(D)** The interactome network of HP+STR-specific binding partners (177 nodes, 649 edges, and 32 orphan nodes). **(E)** The average path length of mPFC-specific (left panel, green line) and HP+STR-specific (right panel, red line) interactome networks are shorter than the mean values (black dotted lines) of random networks from the mouse brain interactome. **(F)** The degree distributions of mPFC- (green line) and HP+STR-specific (red line) interactome networks follow a power-law decay, P(x) = (x)ˆ(-α+1), where the exponent α is 2.98 and 2.75, respectively. **(G)** The degree densities of mPFC- (green line) and HP+STR-specific (red line) interactome networks.

To test whether and to what extent Shank3 interacts with different groups of proteins in each brain region, we compared the newly generated mPFC Shank3 interactome with the previously generated HP+STR interactome (**Figure 2A** and Supplementary Table S1). We found that, unexpectedly, only 47 proteins (including Shank3) were common between the mPFC (22.3% of 211 proteins) and HP+STR (18.4% of 255 proteins) interactomes, while 164 and 208 proteins were specifically identified from the mPFC (77.7%) and HP+STR (81.6%) interactomes, respectively. There were smaller overlaps between the Shank3 Y2H screening and either of the *in vivo* interactomes (11 and 14 proteins for the mPFC and HP+STR interactomes, respectively) (**Figure 2A** and Supplementary Table S1), which might be due to the methodological differences between Y2H and IP. While Y2H is more suitable for detection of transient but direct interactions (Perkins et al., 2010), IP is useful to identify protein complexes.

Next we constructed interactome networks of the common (47), mPFC-specific (164), and HP+STR-specific (208) Shank3 interactors using the PPI data from the Agile Protein Interactomes DataServer (APID) (Alonso-Lopez et al., 2016) (**Figures 2B–D**). From these networks, we noticed that the Homer and DLGAP (also called GKAP/SAPAP) proteins were in the common Shank3 interactome network (**Figure 2B**). Importantly, these proteins are considered to organize the core structure of the post-synaptic density (PSD) by directly interacting with Shank proteins (Chen et al., 2008; Sheng and Kim, 2011).

Supporting the strong connectivity of networks, the average path length of either the mPFC- (2.89) or HP+STR-specific (2.27) Shank3 interactome network was shorter than those of the networks comprising the same number of proteins randomly selected from the mouse brain interactome (mean values of 3.87 and 4.32 for the mPFC- and HP+STR-specific interactome, respectively) (see Materials and Methods) (**Figure 2E**). Moreover, the degree distributions of mPFC- and HP+STR-specific Shank3 interactomes followed a power-law decay (**Figure 2F**). When we calculated the degree distributions, the value for the HP+STR-specific interactome network was slightly higher than that for the mPFC-specific interactome network (**Figure 2G**).

### Comparisons of Brain Region-specific Shank3 Interactomes with Brain Region Enriched Proteomes

**FIGURE 3:**
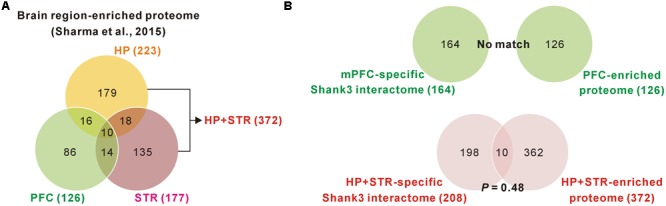
**Comparisons of brain region-specific Shank3 interactomes with the corresponding brain region-enriched proteomes. (A)** The Venn diagram shows the number of brain-region enriched proteins for the PFC, HP, and STR. The data was obtained from a large-scale quantitative proteomic analysis on mouse brain regions (Sharma et al., 2015). **(B)** The Venn diagram shows the number of common proteins between the brain region-specific Shank3 interactomes and the corresponding brain region-enriched proteins. There was no common protein for the mPFC-specific Shank3 interactome (upper panel), while 10 proteins were common for the HP+STR-specific Shank3 interactome, which was not statistically significant (*P* = 0.48, hypergeometric test) (lower panel).

We found a ∼20% overlap of protein identities between the mPFC and HP+STR Shank3 interactomes. We next investigated what might cause the large difference between the mPFC and HP+STR Shank3 interactomes. One possibility is that the mPFC and HP+STR tissues might have different proteome expression profiles which Shank3 can interact with. However, a recently reported large-scale quantitative proteomic analysis on the 10 major regions of the mouse brain showed that the PFC, HP, and STR were clustered more tightly in term of their proteome expression profiles compared to the other brain regions (Sharma et al., 2015). In the same study, nevertheless, they also identified 2,901 brain region-enriched proteins, defined by a >fourfold expression level in a specific brain region over their median abundance across the other regions. Based on these data, we found that there were 126, 223, and 177 proteins enriched in the PFC, HP, and STR (372 for HP+STR), respectively (**Figure 3A**). We compared these protein lists with the corresponding mPFC- (164) and HP+STR-specific (208) Shank3 interactomes to understand whether there was a significant number of brain region-enriched proteins in each interactome. However, we found that there was no PFC-enriched protein in the mPFC-specific Shank3 interactome, and there were only 10 HP+STR-enriched proteins in the HP+STR-specific Shank3 interactome (*P* = 0.48, hypergeometric test) (**Figure 3B**). Taken together, these results suggest that the large difference between mPFC and HP+STR Shank3 interactomes could not be caused by differences in the protein expression profiles among the brain regions.

### Pathway Analysis of Common and Brain Region-specific Shank3 Interactomes

**FIGURE 4:**
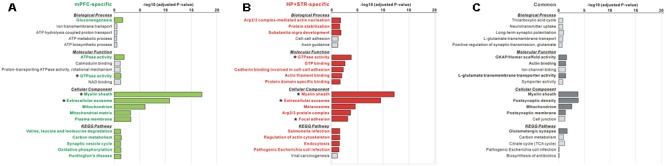
**Gene Ontology (GO) and Kyoto Encyclopedia of Genes and Genomes (KEGG) pathway analysis of common and brain region-specific Shank3 interactomes. (A)** GO and KEGG pathway analysis of the mPFC-specific Shank3 interactome (164 proteins). Significant terms (Benjamini adjusted *P*-value < 0.05) are written in bold green. **(B)** GO and KEGG pathway analysis of the HP+STR-specific Shank3 interactome (208 proteins). Significant terms are written in bold red. **(C)** GO and KEGG pathway analysis of the common Shank3 interactome from the mPFC and HP+STR (47 proteins). Significant terms are written in bold gray. Asterisks indicate the terms commonly significant in both the mPFC- and HP+STR-specific interactomes. Only the top five terms of each category are shown in the graphs. The full results of the pathway analysis are presented in Supplementary Tables S2–S4.

The large difference in the protein identities between the mPFC and HP+STR Shank3 interactomes prompted us to investigate whether the biological pathways represented by the two interactomes are also different. Even though the protein identities were different, it is also possible that they could be participating in the same or similar pathways. To understand this, we performed GO and KEGG pathway analysis of the Shank3 interactors identified specifically in the mPFC or HP+STR (164 and 208 proteins, respectively) interactome. For the mPFC-specific Shank3 interactome, terms including “gluconeogenesis” in the biological process (BP) category, “ATPase activity” and “GTPase activity” in the molecular function (MF) category, “myelin sheath,” “extracellular exosome,” and “mitochondrion” in the cellular component (CC) category, and “valine, leucine, and isoleucine degradation,” “carbon metabolism” and “synaptic vesicle cycle” in the KEGG pathway category were observed to be significant (**Figure 4A** and Supplementary Table S2). Meanwhile, for the HP+STR-specific Shank3 interactome, terms including “Arp2/3 complex-mediated actin nucleation,” “protein stabilization,” and “substantia nigra development” in the BP category, “GTPase activity,” “cadherin binding involved in cell-cell adhesion,” and “actin filament binding” in the MF category, “myelin sheath,” “extracellular exosome,” “Arp2/3 protein complex,” and “focal adhesion” in the CC category, and “regulation of actin cytoskeleton” and “endocytosis” in the KEGG pathway were observed to be significant (**Figure 4B** and Supplementary Table S3). Therefore, most of the significant terms from the mPFC- and HP+STR-specific Shank3 interactomes did not overlap, except for some broad terms in the MF and CC categories such as “GTPase activity” and “extracellular exosome.” These results suggest that the representative or major biological functions of the brain region-specific Shank3 interactomes could be different.

Next, we also performed GO and KEGG pathway analysis for the 47 common Shank3 interactors from the mPFC and HP+STR interactomes. We found that the terms including “GKAP/Homer scaffold activity” and “actin binding” in the MF category, “myelin sheath,” “post-synaptic density,” and “mitochondrion” in the CC category, and “glutamatergic synapse” in the KEGG pathway were significant (**Figure 4C** and Supplementary Table S4). These results were consistent with our observation that the Homer and GKAP/SAPAP proteins, Shank3-interacting core scaffolds of the PSD, were identified in the common interactome (**Figure 2B**). There was no significant term in the BP category, possibly due to the small number of proteins in the common interactome.

### Disease Associations of Brain Region-specific Shank3 Interactomes

Recent studies have shown that genes mutated in the same type of neurodevelopmental or neuropsychiatric disorder such as ASD or SCZ could be highly interconnected through the PPI networks (O’Roak et al., 2012; De Rubeis et al., 2014; Fromer et al., 2014). Therefore, considering the strong association of *SHANK3* with multiple neurodevelopmental and neuropsychiatric disorders, we investigated whether there were also a significant number of disease-associated proteins in the mPFC- and HP+STR-specific Shank3 interactomes. To compare with the Shank3 interactomes, we selected three established disease-associated gene lists; intellectual disability-associated FMRP (Fragile X mental retardation protein) target genes (Darnell et al., 2011), ASD-associated SFARI (Simons Foundation Autism Research Initiative) genes^4^, and psychiatric disorder-associated PsyGeNET (Psychiatric disorders Gene association NETwork) genes (Gutierrez-Sacristan et al., 2015).

**FIGURE 5:**
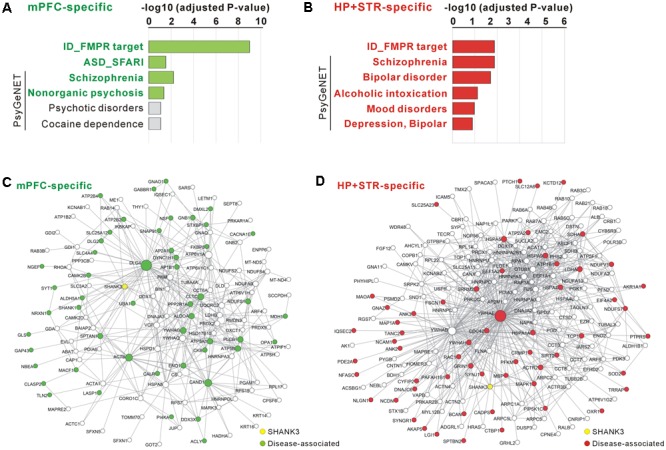
**Disease associations of the mPFC- and HP+STR-specific Shank3 interactomes. (A)** Disease association analysis of the mPFC-specific Shank3 interactome. Significant disease terms (Benjamini adjusted *P*-value < 0.05) are written in bold green. **(B)** Disease association analysis of the HP+STR-specific Shank3 interactome. Significant disease terms are written in bold red. Only the top six disease terms are shown in the graphs. The full results of the analysis are presented in Supplementary Tables S5, S6. ID, intellectual disability. **(C)** The mPFC-specific Shank3 interactome network where the disease-associated protein nodes are colored in green. **(D)** The HP+STR-specific Shank3 interactome network where the disease-associated protein nodes are colored in red.

For the mPFC-specific Shank3 interactome, there were 41 FMRP target genes (adjusted *P* = 9.70E-10, hypergeometric test), nine SFARI genes (*P* = 0.03), and 21 SCZ-associated genes from the PsyGeNET database (*P* = 0.006) (**Figure 5A** and Supplementary Table S5). For the HP+STR-specific Shank3 interactome, there were 36 FMRP target genes (*P* = 0.0002), 29 SCZ- and 19 BD-associated PsyGeNET genes (*P* = 0.0002 and 0.0004, respectively) (**Figure 5B** and Supplementary Table S6). However, there was no SFARI gene in the HP+STR-specific Shank3 interactome. In the mPFC- and HP+STR-specific interactome networks, these disease-associated proteins were highly interconnected with other proteins (**Figures 5C,D**). Taken together, these results suggest that the brain region-specific Shank3 interactomes could be a useful platform for understanding the disease associations as well as synaptic functions of Shank3.

## Discussion

Shank family proteins are one of the most abundant proteins in neuronal excitatory synapses (Sheng and Hoogenraad, 2007). Together with other abundant scaffold proteins (such as PSD-95, GKAP/SAPAP, and Homer), Shank organizes the core structural framework of the PSD (Baron et al., 2006; Chen et al., 2008; Sheng and Kim, 2011), at which 100s of other synaptic molecules communicate and function. Therefore, the heterogeneity of neuronal pathophysiology related to *SHANK3* mutations might be, at least partly, explained by the diversity of Shank3-interacting proteins in different brain regions, which has not been directly investigated yet.

**FIGURE 6:**
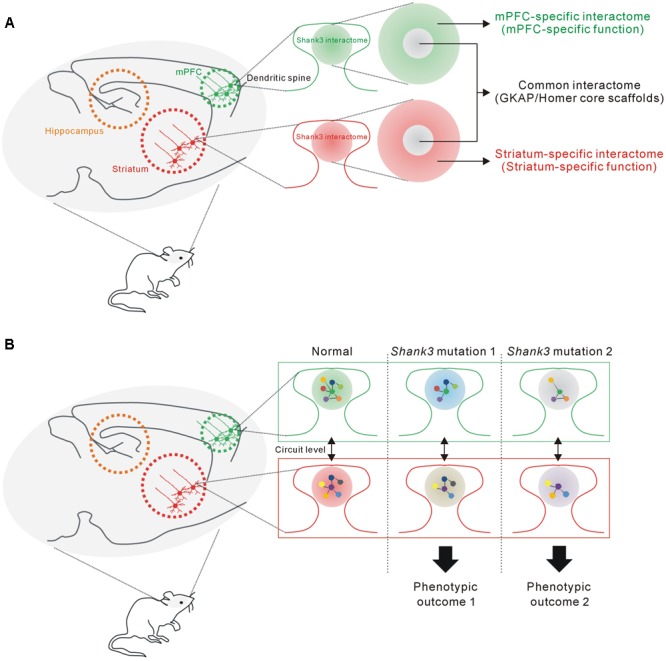
**The proposed hypothesis that the Shank3 interactome in each brain region consists of common and region-specific interactors, and how this could contribute to the phenotypic diversity of *SHANK3* mutations. (A)** The proposed model suggesting that the Shank3 interactome in each brain region (e.g., mPFC or striatum) consists of two parts. The core part contains common interactors such as GKAP and Homer scaffolds that mainly mediate the structural functions of Shank3 at the PSD. The remaining interactors, which constitute a majority of the proteins, contribute to the brain region-specific functions of Shank3. **(B)** The proposed hypothesis suggests how the brain region-specific Shank3 interactomes could contribute to the phenotypic diversity related to *SHANK3* mutations (see main text for details).

In this study, we found that the mPFC and HP+STR Shank3 interactomes were largely different, having only 20% of the interacting proteins in common. Moreover, GO and KEGG pathway analysis of the mPFC- and HP+STR-specific Shank3 interactomes indicated that their major functional and biological pathways were also different. Meanwhile, “scaffold activity” and “post-synaptic density” were revealed as the representative pathways of common Shank3 interactors which include Homer and GKAP/SAPAP core scaffolds of the PSD. From these results, we propose a model that the Shank3 interactome in each brain region could consist of two different parts (**Figure 6A**). The core part contains direct, strong, and thus, likely common interactors of Shank3 such as Homer and GKAP/SAPAP, which mainly mediate the structural role of Shank3 in the PSD. In contrast, rest of the proteins in each interactome (which constitute the majority) form a complex with Shank3 in a brain region-specific manner, thereby allowing the functional diversity of Shank3. The role of Shank3 in a specific synapse can be determined by the combined effect from common and region-specific interactors, which could underlie the fine-tuning of Shank3-mediated synaptic development and function. Indeed, recent studies of *Shank3* KO mice demonstrated brain region-specific synaptic functions of Shank3 (Peca et al., 2011; Lee et al., 2015; Vicidomini et al., 2016). For example, in the *Shank3Δ11^-/-^* mice, synaptic localization and interaction of Homer and metabotropic glutamate receptor 5 (mGluR5), and mGluR5-dependent signaling are altered in the striatum and cortex, but not in the hippocampus (Vicidomini et al., 2016). Further characterization of the Shank3 interactomes and their synaptic functions from more brain regions is necessary to confirm this intriguing yet premature hypothesis.

Regarding the pathway analysis, some broad terms in the MF and CC categories such as “GTPase activity,” “myelin sheath,” and “extracellular exosome” were commonly significant from the mPFC- and HP+STR-specific Shank3 interactomes. Importantly, recent studies have revealed physical and functional associations between Shank3 and various GTPases. For example, Shank3 interacts with Rho-GAP interacting CIP4 homolog 2 (Rich2), which is involved in regulating AMPA receptor trafficking (Raynaud et al., 2013) and dendritic spine morphology by modulating the activities of Rac1 and Cdc42 (Sarowar et al., 2016b). Moreover, Shank3 directly interacts with Rap1 and R-Ras via the Shank/ProSAP N-terminal (SPN) domain to control their membrane localization (Lilja et al., 2017). At this moment, it is not easy to explain the roles of Shank3 in myelin sheath, but we could find the expression of Shank3 transcripts in the myelinating oligodendrocyte from a publicly available RNA sequencing database^5^ (Zhang et al., 2014). Investigating whether Shank3 could have cell type-specific functions and whether this could be mediated by differential interactors of each cell type will be interesting future directions.

Notably, there was no significant overlap between the mPFC- or HP+STR-specific Shank3 interactome and the corresponding brain region-enriched proteomes. Therefore, it is unlikely that the large difference between the mPFC and HP+STR Shank3 interactomes is due to differences in the protein expression profiles among the brain regions. Instead, other possibilities such as region-specific differential expression of Shank3 isoforms (Wang et al., 2014) or post-translational modifications of the synaptic proteome might have larger effects on the interactomes. Since the post-translational modifications of Shank3 are poorly defined, mass spectrometry-based approaches on the IP-enriched Shank3 proteins from different brain regions will be very informative; in this regard, the *EGFP-Shank3* TG mice could prove to be a useful tool.

Importantly, we found a significant number of neurodevelopmental and neuropsychiatric disorder-associated proteins in the mPFC- and HP+STR-specific Shank3 interactomes. This result is consistent with the results from recent studies showing that groups of genes mutated in ASD or SCZ could generate highly interconnected PPI networks (O’Roak et al., 2012; De Rubeis et al., 2014; Fromer et al., 2014). Based on this idea, it is tempting to speculate that other proteins in the Shank3 interactomes might be potential candidates for the novel risk genes for neurodevelopmental and neuropsychiatric disorders. Studies on the synaptic functions and relationships of some proteins with Shank3, especially the hub proteins in the Shank3 interactome networks, could be an interesting future direction.

How can the brain region-specific Shank3 interactomes contribute to the phenotypic diversity of *SHANK3* mutations? First, the Shank3 interactome and synaptic functions mediated by the interactome of a specific brain region (e.g., mPFC) might be differentially affected by various *SHANK3* mutations, if the mutations target distinct PPI domains. Second, the interactomes and synaptic properties of different brain regions (e.g., mPFC and STR) might be differentially affected by a single *SHANK3* mutation, as the interactomes consist of largely different groups of proteins. Lastly, these brain regions are connected by neural circuits and thus the phenotypic, usually behavioral, outcome of each *SHANK3* mutation is determined by the combined effect of synaptic changes in multiple brain regions (**Figure 6B**).

One of the limitations of current study is that our mPFC and HP+STR Shank3 interactomes were not generated in parallel. Indeed, there are many factors that could affect interpretation of the results, including a few differences in methods of mass spectrometry analysis, and difference in mouse strains (C57BL/6J and FVB/N for mPFC and HP+STR interactome, respectively). More importantly, IP and mass spectrometry analysis were performed only once to produce each interactome list. Therefore, repetition of the experiments with consistent methods is required to confirm the results of current study. In addition, it is not easy to define direct or indirect interactions from our *in vivo* interactomes. However, the small overlaps between Shank3 Y2H screening and either of the *in vivo* interactomes suggest that majority of the proteins could be indirect binding partners of Shank3. Direct validation for each interaction of Shank3 is required to address this issue.

Another limitation is that our mPFC and HP+STR Shank3 interactomes are only snapshots of Shank3 interactions during the steady state. Like many known protein interactions at the neuronal synapse, however, the interactions between Shank3 and other proteins could be dynamically regulated by many factors such as developmental stages and neuronal activity. For example, the expression levels of Shank3 isoforms are modulated during brain development and by neuronal depolarization (Wang et al., 2014). As each Shank3 isoform contains different PPI domains, the Shank3 interactome might be affected when the isoform expressions levels are altered. Although little is known about the post-translational modifications of Shank3, neuronal activity or synaptic plasticity might induce phosphorylation of Shank3 and its interacting proteins, thereby modulating their interaction. In addition, zinc ion that binds to the SAM domain of Shank3 might affect the interactome by regulating the structural assembly and synaptic localization of Shank3 (Baron et al., 2006; Grabrucker et al., 2014; Tao-Cheng et al., 2016). Furthermore, Shank3 levels could be regulated by various physiological conditions including circadian rhythm (Sarowar et al., 2016a), which can also affect Shank3 interactome. More comprehensive and quantitative analysis of the Shank3 interactome in both physiological and pathological conditions will help us better understand its dynamic regulation and potential implications for various brain disorders.

## Conclusion

Our study provides evidence that Shank3 can form protein complexes in a brain region-specific manner, which further expands our understanding of the heterogeneity and complexity of *SHANK3*-related brain disorders.

## Author Contributions

YL, BL, YZ, YK, SK, and KH designed and performed the experiments. HK and KH analyzed and interpreted the data. W-KK discussed the project and provided reagents. YL, HK, and KH wrote the paper. All authors read and approved the manuscript.

## Conflict of Interest Statement

The authors declare that the research was conducted in the absence of any commercial or financial relationships that could be construed as a potential conflict of interest.

## Footnotes

1 http://cicblade.dep.usal.es:8080/APID/init.action#tabr1

2 http://www.genenames.org/

3 https://gene.sfari.org/autdb/GS_Home.do

4 https://gene.sfari.org/autdb/GS_Home.do

5 http://web.stanford.edu/group/barres_lab/brain_rnaseq.html

## Supplementary Material

FIGURE S1 **Detection of synaptic proteins, but not a nuclear protein, in P2 crude synaptosomal fraction.** Western blot images show PSD-95 and Shank3 in P2 fraction. Nuclear protein, NeuN, was not detected in P2 fraction. H, homogenates; P1, nuclei and other large debris; P2, crude synaptosomes; S2, supernatant after P2 precipitation.

FIGURE S2 **Normal expression levels of synaptic proteins in the mPFC of *Shank3* TG mice.** Representative Western blot images and quantification show normal expression levels of some synaptic proteins in the mPFC of Shank3 TG mice. Data are presented as mean ± SEM (*n* = 4 animals per genotype, unpaired two-tailed Student’s *t*-test).xs
