# Supplementary figures and images for: Integrative Analysis of Brain Region-specific Shank3 Interactomes for Understanding the Heterogeneity of Neuronal Pathophysiology Related to SHANK3 Mutations
Source: Front Mol Neurosci. 2017 Apr 19;10:110. doi: 10.3389/fnmol.2017.00110 (PMC5395616; doi:10.3389/fnmol.2017.00110)

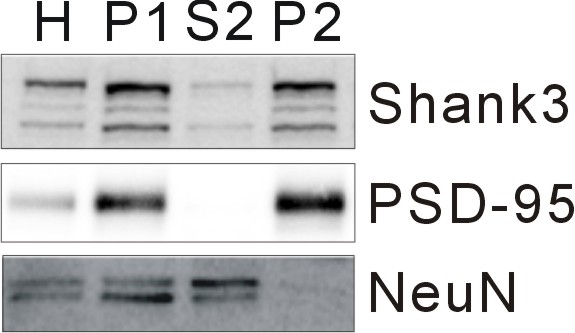

Supplement: FIGURE S1 — Detection of synaptic proteins, but not a nuclear protein, in P2 crude synaptosomal fraction. Western blot images show PSD-95 and Shank3 in P2 fraction. Nuclear protein, NeuN, was not detected in P2 fraction. H, homogenates; P1, nuclei and other large debris; P2, crude synaptosomes; S2, supernatant after P2 precipitation. [file Image_1.JPEG]

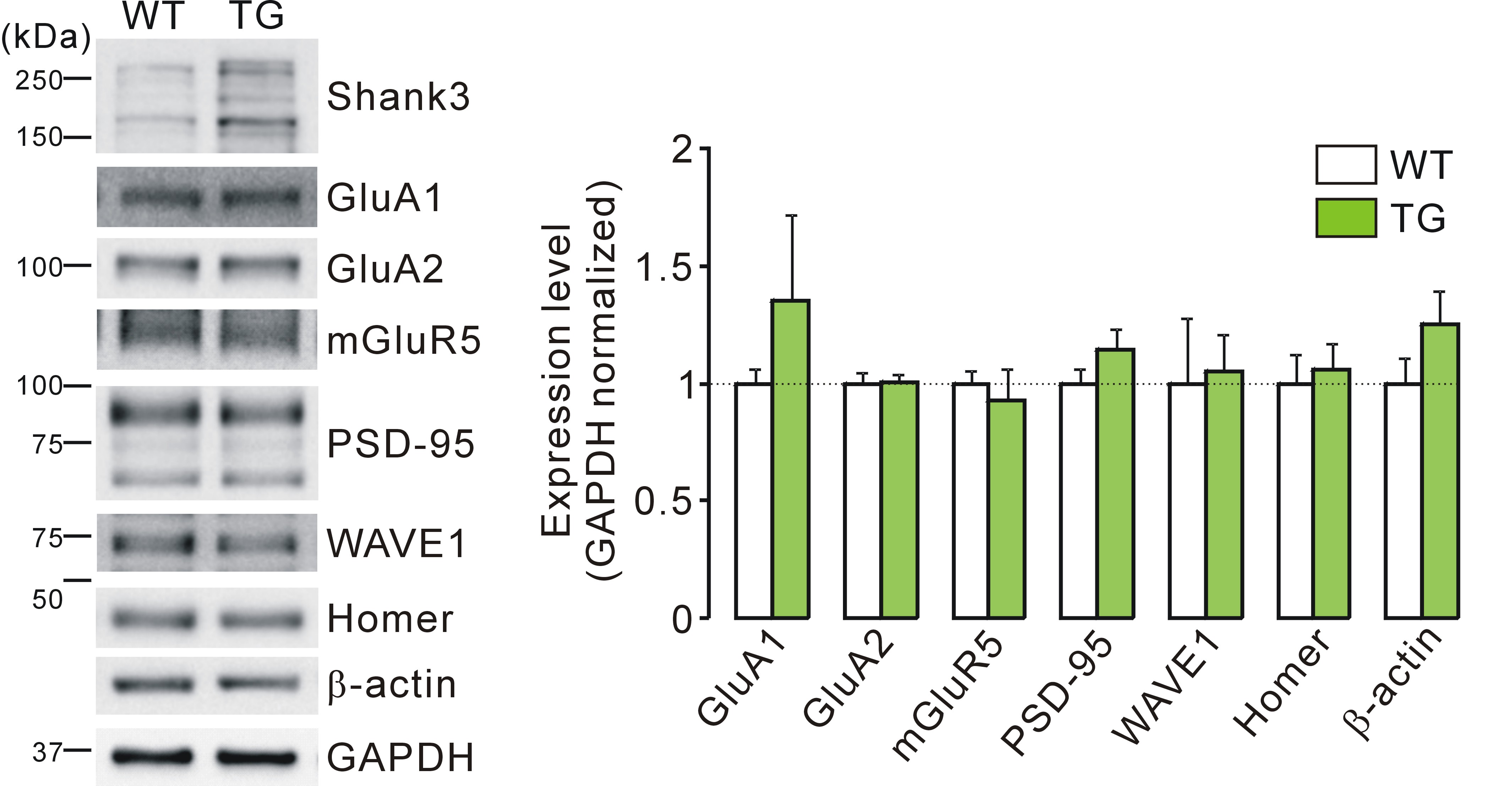

Supplement: FIGURE S2 — Normal expression levels of synaptic proteins in the mPFC of Shank3 TG mice. Representative Western blot images and quantification show normal expression levels of some synaptic proteins in the mPFC of Shank3 TG mice. Data are presented as mean ± SEM (n = 4 animals per genotype, unpaired two-tailed Student’s t-test).xs [file Image_2.JPEG]
